# Assessment of individual molecular response in chronic myeloid leukemia patients with atypical *BCR-ABL1* fusion transcripts: recommendations by the EUTOS cooperative network

**DOI:** 10.1007/s00432-021-03569-8

**Published:** 2021-03-07

**Authors:** Vivien Schäfer, Helen E. White, Gareth Gerrard, Susanne Möbius, Susanne Saussele, Georg-Nikolaus Franke, François-X. Mahon, Rodica Talmaci, Dolors Colomer, Simona Soverini, Katerina Machova Polakova, Nicholas C. P. Cross, Andreas Hochhaus, Thomas Ernst

**Affiliations:** 1 Abteilung Hämatologie/Onkologie, Klinik für Innere Medizin II, Universitätsklinikum Jena, Am Klinikum 1, 07747 Jena, Germany; 2 Wessex Regional Genetics Laboratory, Salisbury NHS Foundation Trust, Salisbury, UK; 3 Faculty of Medicine, Imperial College London, London, UK; 4 III. Medizinische Klinik, Medizinische Fakultät Mannheim der Universität Heidelberg, Mannheim, Germany; 5 Department of Hematology and Oncology, University of Leipzig, Leipzig, Germany; 6 Bergonie Institute Cancer Center Bordeaux, INSERM U1218, University of Bordeaux, Bordeaux, France; 7 Hematology Department, Fundeni Clinical Institute, University of Medicine and Pharmacy ‘Carol Davila’, Bucharest, Romania; 8 Hematopathology Unit, Department of Pathology, University of Barcelona, Barcelona, Spain; 9 Department of Experimental, Diagnostic and Specialty Medicine, Institute of Hematology “Lorenzo e Ariosto Seràgnoli”, University of Bologna, Bologna, Italy; 10 Department of Molecular Genetics, Institute of Hematology and Blood Transfusion, Prague, Czech Republic; 11 School of Medicine, University of Southampton, Southampton, UK

**Keywords:** Chronic myeloid leukemia, CML, *BCR-ABL1*, Atypical transcripts, Molecular monitoring

## Abstract

**Purpose:**

Approximately 1**–**2% of chronic myeloid leukemia (CML) patients harbor atypical *BCR-ABL1* transcripts that cannot be monitored by real-time quantitative PCR (RT-qPCR) using standard methodologies. Within the European Treatment and Outcome Study (EUTOS) for CML we established and validated robust RT-qPCR methods for these patients.

**Methods:**

*BCR-ABL1* transcripts were amplified and sequenced to characterize the underlying fusion. Residual disease monitoring was carried out by RT-qPCR with specific primers and probes using serial dilutions of appropriate *BCR-ABL1* and *GUSB* plasmid DNA calibrators. Results were expressed as log reduction of the *BCR-ABL1/GUSB* ratio relative to the patient-specific baseline value and evaluated as an individual molecular response (IMR).

**Results:**

In total, 330 blood samples (2–34 per patient, median 8) from 33 CML patients (19 male, median age 62 years) were analyzed. Patients expressed seven different atypical *BCR-ABL1* transcripts (e1a2, *n* = 6; e6a2, *n* = 1; e8a2, *n* = 2; e13a3, *n* = 4; e14a3, *n* = 6; e13a3/e14a3, *n* = 2; e19a2, *n* = 12). Most patients (61%) responded well to TKI therapy and achieved an IMR of at least one log reduction 3 months after diagnosis. Four patients relapsed with a significant increase of *BCR-ABL1/GUSB* ratios.

**Conclusions:**

Characterization of atypical *BCR-ABL1* transcripts is essential for adequate patient monitoring and to avoid false-negative results. The results cannot be expressed on the International Scale (IS) and thus the common molecular milestones and guidelines for treatment are difficult to apply. We, therefore, suggest reporting IMR levels in these cases as a time-dependent log reduction of *BCR-ABL1* transcript levels compared to baseline prior to therapy.

**Supplementary Information:**

The online version contains supplementary material available at 10.1007/s00432-021-03569-8.

## Introduction

Chronic myeloid leukemia (CML) is characterized by the Philadelphia chromosome (Ph), produced by the balanced reciprocal translocation t(9;22)(q34;q11) leading to the breakpoint cluster region-Abelson (*BCR-ABL1*) fusion gene (Hehlmann et al. 2007). This oncogene is translated into the chimeric *BCR-ABL1* protein with constitutive tyrosine kinase activity which results in reduced apoptosis, deregulated cell proliferation and decreased differentiation of hematopoietic progenitors (Quintás-Cardama and Cortes 2009).

More than 95% of Ph^+^ CML patients express the typical e13a2 (b2a2) and/or e14a2 (b3a2) *BCR-ABL1* transcripts located within the major breakpoint cluster region (M-*BCR*) associated with a p210 fusion protein. Overall, three breakpoint regions are present in the *BCR* gene whereby the M-*BCR* spans from exon 12 to exon 16 (formerly named exons b1–b5). The most frequent breakpoint region of the *ABL1* gene is located at a 200 kb segment upstream of exon 2 (a2) (Ross et al. 2013). Nevertheless, 1–2% of CML patients have rearrangements outside the M-*BCR* or with another *ABL1* exon (Cayuela et al. 2005; Baccarani et al. 2019). For instance, the transcript e1a2 with the breakpoint in the minor breakpoint cluster region (m-*BCR*) producing a p190 protein was found initially in two-thirds of Ph^+^ acute lymphoblastic leukemia (ALL) patients. A breakpoint in the micro breakpoint cluster region (µ-*BCR*) leads to the largest chimeric *BCR-ABL1* with an e19a2 junction translated into a p230 protein product (Melo 1997). Several other uncommon fusion transcripts in CML have been identified so far such as e6a2 (Hochhaus et al. 1996) or e8a2 (Branford et al. 2000) as well as transcripts missing the *ABL1* exon a2 [e.g. e13a3 (b2a3) or e14a3 (b3a3)].

The characterization of the precise rearrangement at diagnosis with a qualitative multiplex PCR and sequencing (Cross et al. 1994) is of critical importance for subsequent monitoring of residual disease and assessment of treatment response (Hochhaus et al. 2020). Thus far, reference material only exists for common transcripts and therefore CML patients with atypical *BCR-ABL1* subtypes remain non-standardised (Yu et al. 2017). Within the European Treatment and Outcome Study (EUTOS) for CML we sought to set up and validate robust qPCR methods for each of these atypical *BCR-ABL1* transcripts. Furthermore, since the levels of disease for these cases cannot be expressed on the International Scale (IS), we suggest a new evaluation criterion for molecular monitoring of these patients—the individual molecular response (IMR) level based on a log reduction from pretreatment levels.

## Materials and methods

### Patients and samples

Peripheral blood samples were obtained from 33 patients (19 male, median age 62 years) with atypical *BCR-ABL1* fusions from nine prospective studies and outside of clinical trials after written informed consent (Table 1 and supplemental table S1). A total of 330 samples (2–34 per patient; median, 8) of different time points were analyzed. Patients expressed seven different atypical *BCR-ABL1* transcripts (e1a2, *n* = 6; e6a2, *n* = 1; e8a2, *n* = 2; e13a3, *n* = 4; e14a3, *n* = 6; e13a3 and e14a3, *n* = 2; e19a2, *n* = 12).

**Table 1:** Patients’ characteristics

	Total	(*n* = 33)
Gender, *n* (%)		
Male	19	(57.6)
Female	9	(27.3)
Unknown	5	(15.2)
Age (years), median (range)	62	(28–78)
*BCR-ABL1* transcript type, *n* (%)		
e19a2	12	(36.4)
e1a2	6	(18.2)
e14a3	6	(18.2)
e13a3	4	(12.1)
e13a3/e14a3	2	(6.1)
e8a2	2	(6.1)
e6a2	1	(3.0)

### RNA extraction and cDNA synthesis

Freshly isolated leukocytes (1 × 10^7^ cells) from peripheral EDTA blood samples were lysed in 1 mL TRIzol^®^ (Invitrogen GmbH, Karlsruhe, Germany) and stored at −20 °C until RNA extraction. Frozen samples were allowed to thaw at room temperature and RNA was isolated using the acid guanidinium thiocyanate-phenol–chloroform extraction method. Reverse transcription of 7.7 µL total RNA (maximum 4 µg of RNA) was performed immediately after isolation with random hexamer primer and M-MLV reverse transcriptase (Invitrogen GmbH) for 60 min at 37 °C according to the manufacturer’s recommendations.

### Multiplex PCR

For identification of atypical *BCR-ABL1* fusion genes multiplex PCR was performed as designed by Cross et al*.* (1994) which uses four oligonucleotide primers (BCR-C: ACCGCATGTTCCGGGACAAAAG, B2B: ACAGAATTCCGCTGACCATCAATAAG, C5e^−^: ATAGGATCCTTTGCAACCGGGTCTGAA, CA3^−^: TGTTGACTGGCGTGATGTAGTTGCTTGG). PCR was carried out with a master mix containing: 1.2 × Taq polymerase buffer with MgCl_2_ (Qiagen, Hilden, Germany)_,_ 0.24 mM desoxynucleoside triphosphate (dNTPs) mix (ThermoFisher Scientific, Darmstadt, Germany), 0.6 µM of each primer (Eurofins Genomics GmbH, Ebersberg, Germany), 0.2 mM MgCl_2_ (Qiagen) and 0.03 U/µL *Taq* Polymerase (Jena Bioscience, Jena, Germany). After adding 1 µL of cDNA to 19 µL PCR mix, the reaction was performed using a thermocycler (Eppendorf, Hamburg, Germany) with the following conditions: initial denaturation at 96 °C for 2 min, 35 cycles of 96 °C for 45 s, 60 °C for 30 s, 72 °C for 50 s and final extension at 72 °C for 10 min. Finally, reaction products were electrophoresed on a 3% agarose gel for detection of the *BCR-ABL1* transcript. As controls for multiplex PCR the cell lines SD1 (e1a2 *BCR-ABL1*^+^ ALL cell line), K562 (e14a2 *BCR-ABL1*^+^ CML blast crisis cell line) and BV173 (e13a2 *BCR-ABL1*^+^ CML blast crisis cell line) were used.

### Direct sequencing

All patient samples with an unusual PCR band on multiplex PCR analysis were characterized by Sanger sequencing and confirmed to have atypical *BCR-ABL1* fusions. Therefore, cDNA was amplified with oligonucleotides for the respective atypical *BCR-ABL1* transcript (Table 2). All PCRs were performed using Ampli*Taq*Gold (Life Technologies, Darmstadt, Germany) with the following conditions: initial denaturation for 10 min at 94 °C, 45 cycles of denaturation for 60 s at 94 °C, annealing for 60 s at 60 °C and extension for 60 s at 72 °C followed by the final elongation for 10 min at 72 °C. Purified amplicons were sequenced bidirectionally by standard Sanger sequencing on a 3500 Genetic Analyzer (Life Technologies). Purification was performed as previously described (Rinke et al. 2013) and subsequent sequencing reactions were carried out using the BigDye Terminator Cycle Sequencing Kit v1.1 (Life Technologies) according to the manufacturer’s recommendations. The sequence data analysis was performed using the Mutation Surveyor software (SoftGenetics, State College, PA, USA).

**Table 2:** Oligonucleotides used for Sanger sequencing and RT-qPCR for the specific atypical *BCR-ABL1* transcripts

Name	Primer sequence 5′ to 3’	Location	Transcript	Product size (bp)
E6F2	CAAAGATGCCAAGGATCCAACGACCAAG	BCR exon 6	e6a2	534
E19F1	GGAGGAGGTGGGCATCTACCG	BCR exon 19	e19a2	567
E8F2	ACGGCAGTCCATGACGGTGAAGAAG	BCR exon 8	e8a2	524
B2A	TTCAGAAGCTTCTCCCTGACAT	BCR exon b2	e13a3/e14a3	422/497
BCR12	CAGATCTGGCCCAACGATGG	BCR exon 1	e1a2	539
NA4-	CGGCTCTCGGAGGAGACGTAGA	ABL exon 4	ABL	
GUS10-lc	AGAAACGATTGCAGGGTTTCAC	GUS exon 10	GUS	205
ENR1162	CCGAGTGAAGATCCCCTTTTTA	GUS exon 12	GUS	

### Cloning of quantification standards

PCR products for the e6a2, e8a2, e14a3, and e19a2 transcripts were generated from patient cDNA samples that expressed the relevant fusions using the oligonucleotides shown in Table 3. The PCR products were cloned into the plasmid pCR2.1 vector using the TA Cloning^®^ Kit (Thermo Fisher) following the manufacturer’s instructions to generate pCR2.1_e6a2, pCR2.1_e8a2, pCR2.1_e14a3 and pPCR2.1_e19a2, respectively. Furthermore, fragments of the *BCR*, *GUSB,* and *ABL1* transcripts, 963, 813, and 1803 bp in size, respectively, were generated from K562-derived cDNA and ligated into the vector pCR2.1. Following digestion using *Eco*R I for pCR2.1_BCR, *Xba* I/*Kpn* I for pCR2.1_GUSB and *Spe* I/*Xba* I for pCR2.1_ABL1 the resulting fragments were subcloned into pUC18 vector to create pUC18_BCR_GUSB (4556 bp) and pUC18 BCR_GUSB_ABL1 (6275 bp) backbone plasmids (supplemental figures S1 and S2).

**Table 3:** Oligonucleotides for the generation of atypical *BCR-ABL1* transcripts inserts for cloning

Oligoname	Primer sequence 5′ to 3’	Transcript	Product size (bp)
BCRex6F	CAAAGATGCCAAGGATCCAACGACCAAG	e6a2	1478
ABLex10R	CTTCGTTCTGAGATACTGGATTCCT		
BCRex7-8F	GTCCTCCATGACTTGCTGAAGCACACT	e8a2	973
ABLex5R	TCTTCCACCTCCATGGTGTC		
BCRex13F	TTCAGAAGCTTCTCCCTGACAT	e14a3	1463
ABLex10R	CTTCGTTCTGAGATACTGGATTCCT		
BCRex18F	GGAGGAGGTGGGCATCTACCG	e19a2	1646
ABLex10R	CTTCGTTCTGAGATACTGGATTCCT		

To allow amplification of the target and control gene from the same construct, the transcript fragments were subcloned into the respective control gene backbone plasmids. Plasmids pUC18 BCR_GUS_e6a2, pUC18 BCR_GUS_e19a2 and pUC18 BCR_GUS_ABL_e14a3 were generated by digestion of pCR2.1 constructs with their specific enzymes (*Hin*d III for e14a2, *Hin*d III/*Xba* I for e6a2 and e19a2, see supplemental figures S3, S5 and S6), while plasmid pUC18_BCR_GUSB_e8a2 was created by amplification of e8a2 from pCR2.1_e8a2 with *Sal* I tagged primers (tagged sequence in bold and underlined with the enzyme cutting site indicated by the /):

BCRex7-8F: **CGAGAG/TCGAC**GTCCTCCATGACTTGCTGAAGCACACT, ABLex5R: **CGAGAG/TCGAC**TCTTCCACCTCCATGGTGTC.

The resulting e8a2 PCR product was digested with *Sal* I and cloned into pUC18_BCR_GUSB digested with *Sal* I to create pUC18_BCR_GUSB_e8a2 (supplemental figure S4).

Standard plasmids used for e1a2 measurement were generated as previously described (Müller et al. 2008). Briefly, e1a2 amplicons were generated from SD1 cell line-derived cDNA and cloned into pCR2.1 vector using the TOPO™ TA Cloning Kit. GUSB fragments were generated from K562-derived cDNA using oligonucleotides with an *Xba* I restriction site. Following *Xba* I digestion products were ligated into the pCR2.1 plasmid using T4 ligase generating plasmid pME3 (supplemental figure S7).

To enable the use as qPCR standards the plasmids were linearised, quantified and serially diluted. Restriction enzymes and molecular weights are shown in Table 4.

**Table 4:** Generated plasmids and their respective restriction enzymes used for linearisation

Plasmid	Inserts	Backbone	Size (bp)	Linearisation
pME3	e1a2, GUSB	pCR2.1	5288	*Not* I
pUCe6a2	e6a2, BCR, GUSB	pUC18	6034	*Hin*d III
pUCe8a2	e8a2, BCR, GUSB	pUC18	5529	*Eco*R V
pUCe19a2	e19a2, BCR, GUSB	pUC18	6202	*Hin*d III
pUCe14a3	e14a3, BCR, ABL1, GUSB	pUC18	7738	*Sal* I

### Real-time quantitative polymerase chain reaction (RT-qPCR)

Expression analysis of atypical *BCR-ABL1* transcripts was performed using the LightCycler instrument 1.5 (Roche Diagnostics, Mannheim, Germany). Each 20 µL reaction mix contained 4 µL LightCycler-FastStart DNA Master^PLUS^ HybProbe master mix (Roche Diagnostics), 2 µL cDNA template or plasmid dilution, 0.5 µM forward primer (specific for atypical *BCR-ABL1* transcript; Table 2), 0.5 µM reverse primer (*ABL1* primer NA4-), 0.25 μM of each hybridization probe listed in Table 5 (TIB Molbiol, Berlin, Germany) and 1 µL Uracil-DNA-Glycosidase (UDG; New England Biolabs GmbH, Frankfurt/Main, Germany). Cycler conditions were: 2 min UDG activation at 50 °C, 10 min denaturation at 95 °C, 45 cycles of 60 s at 95 °C, 10 s at 60 °C and 26 s at 72 °C. For quantification, a 5 log series of plasmid dilutions for every atypical *BCR-ABL1* transcript was amplified within the PCR. Beta-glucuronidase (*GUSB*) transcripts were measured as an internal control using the same standard plasmids as for the *BCR-ABL1* measurement.

**Table 5:** Hybridization probes for monitoring atypical *BCR-ABL1* transcripts

Name	Hybridization probe sequence 5′ to 3’	Location
a3-3´HP	LC Red640-AATGGGGAATGGTGTGAAGCCCAAA-phosphate	ABL exon 3
a3-5´HP	TGAAAAGCTCCGGGTCTTAGGCTATAATCA-fluorescine	ABL exon 3
GUS-F	TGATCCAGACCCAGATGGTACTGCT-fluorescine	GUSB exon 11
GUS-LC	LC Red640-TAGCAGACTTTTCTGGTACTCTTCAGTGAACA-phosphate	GUSB exon 11

Every CML patient had his individual baseline value at diagnosis which was calculated as the ratio *BCR-ABL1*/*GUSB*. This baseline value was set as 1 and log-reduction was calculated for every time of investigation so that one log-reduction means IMR1, two log-reductions IMR2 and so on.

## Results

### Detection of atypical BCR-ABL1 transcripts by multiplex PCR

**Fig. 1:**
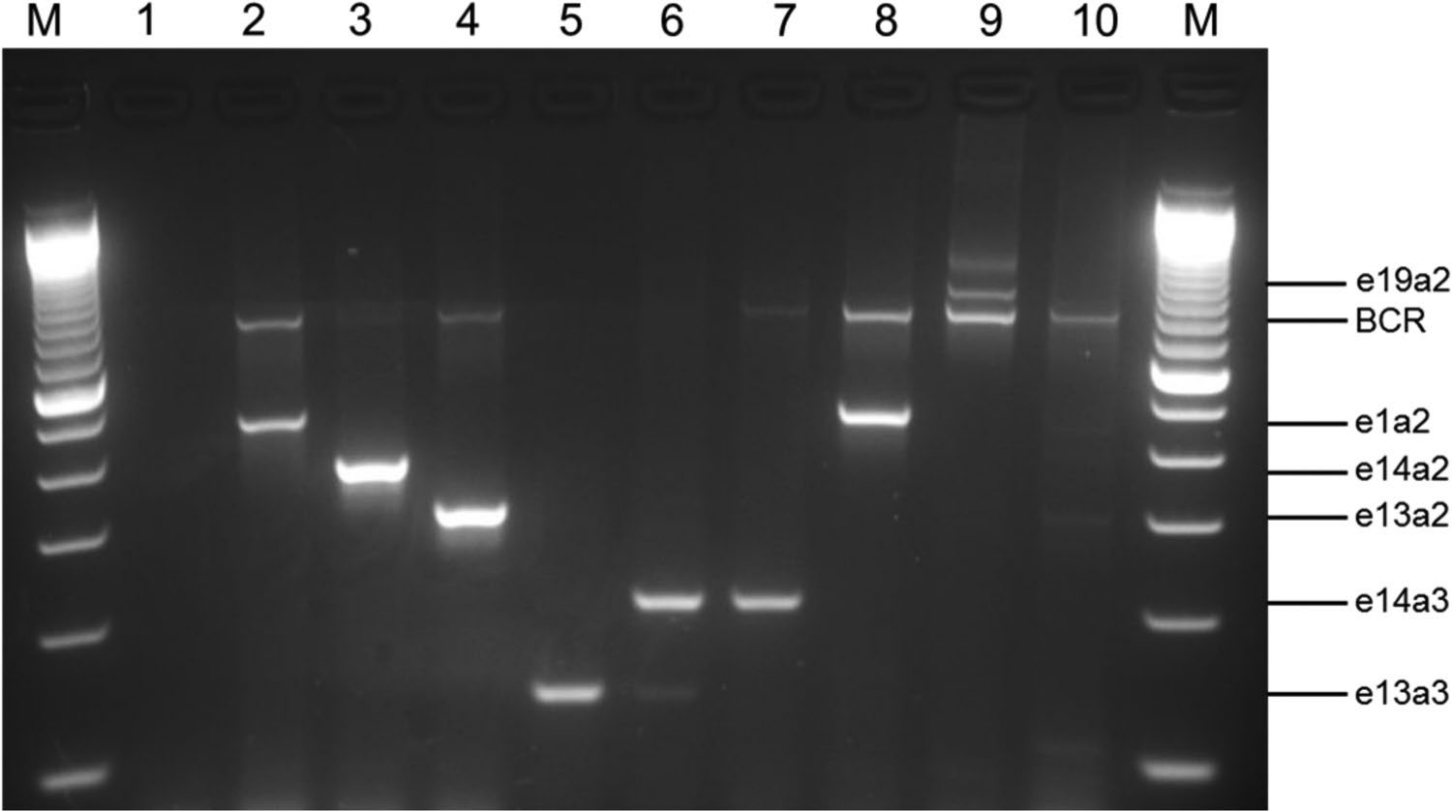
Multiplex PCR for *BCR-ABL1* and *BCR* transcripts. Lane 1, PCR negative control; lane 2, SD1 cell line (e1a2 *BCR-ABL1*, 481 bp); lane 3, K562 cell line (e14a2 *BCR-ABL1*, 385 bp); lane 4, BV173 cell line (e13a2 *BCR-ABL1*, 310 bp); lane 5, e13a3 *BCR-ABL1* CML patient (128 bp); lane 6, e13a3 and e14a3 *BCR-ABL1* CML patient (128 and 203 bp); lane 7, e14a3 *BCR-ABL1* CML patient (203 bp); lane 8, e1a2 *BCR-ABL1* CML patient (481 bp); lane 9, e19a2 *BCR-ABL1* CML patient (925 bp); lane 10, *BCR-ABL1* negative patient; lane M, 100 bp marker. *BCR* bands (808 bp) are an internal positive control for all cell lines and patients

Atypical *BCR-ABL1* fusion genes were determined by multiplex PCR whereby the size of the resulting PCR product depended on the breakpoint in the *BCR* and *ABL1* gene. Figure 1 represents eight different *BCR-ABL1* transcripts detected in patients and cell lines. The typical *BCR-ABL1* transcripts e13a2 and e14a2 are shown by the CML cell lines BV173 and K562 with a PCR product size of 310 bp (lane 4) and 385 bp (lane 3), respectively. The small fusion transcript e13a3 with a product size of 128 bp is found in lane 5 of the agarose gel and one sample with the *BCR-ABL1* double transcript e13a3 and e14a3 (product size 128 and 203 bp) is shown in lane 6. A much greater DNA fragment of 481 bp presents the e1a2 fusion gene found in the ALL cell line SD1 (lane 2) and rarely in CML patients (lane 8). The largest currently known *BCR-ABL1* transcript in CML patients is the e19a2 transcript with a PCR product size of 925 bp (lane 9) which is located above the *BCR* control band. This *BCR* fragment with a size of 808 bp serves as an internal control for the RNA quality and was visible for patients without a *BCR-ABL1* fusion gene (lane 10). All atypical transcripts were verified by Sanger sequencing.

### Monitoring of response to therapy in CML patients with atypical BCR-ABL1 transcripts by RT-qPCR

**Fig. 2:**
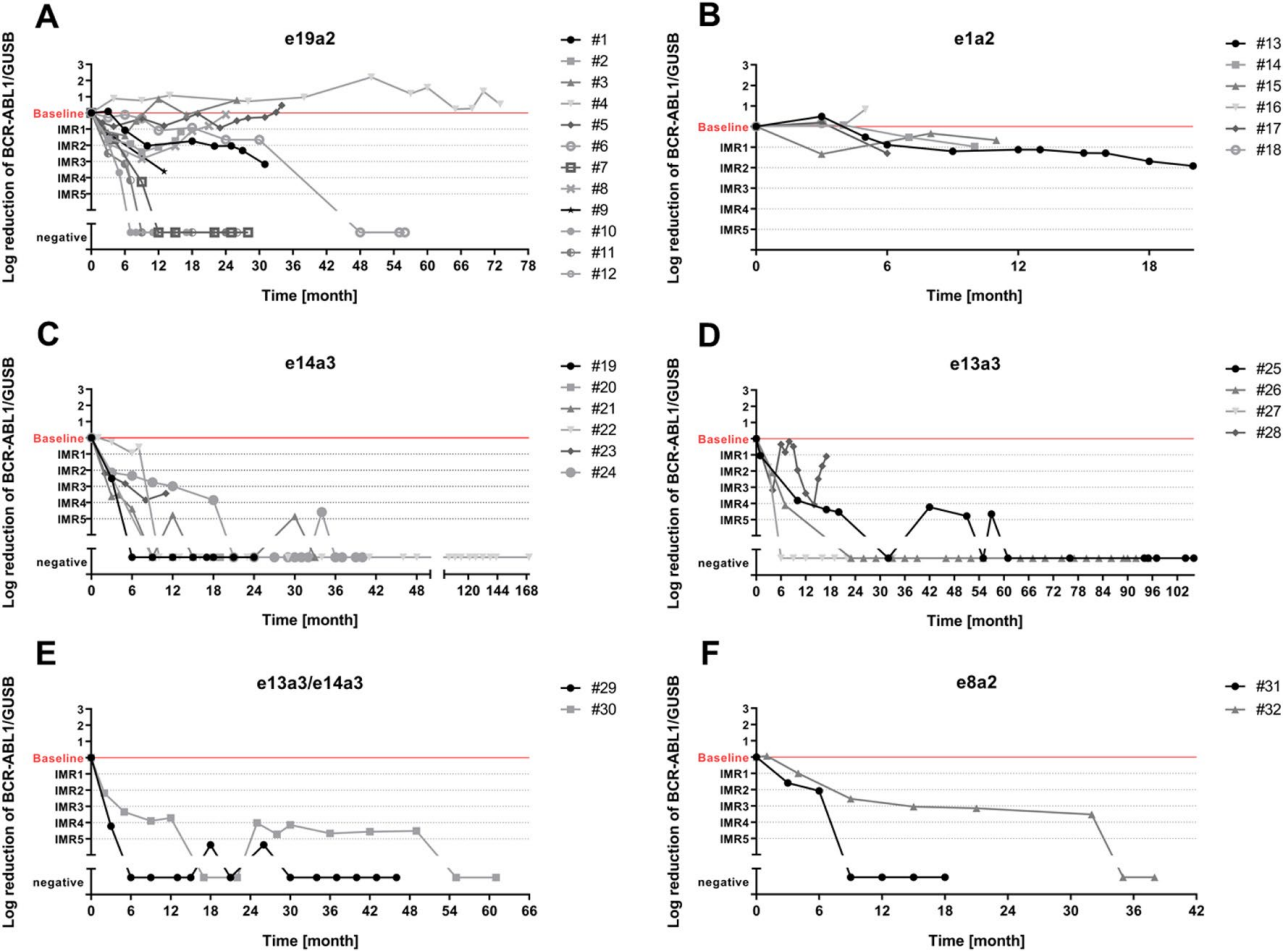
Monitoring of CML patients with atypical *BCR-ABL1* transcripts treated with tyrosine kinase inhibitors by RT-qPCR as log reduction in relation to the individual baseline value. The molecular response to therapy was evaluated as individual molecular response (IMR) levels. **a** Patients with e19a2 *BCR-ABL1* transcript (*n* = 12) were monitored in the median for 17 months (range 9–73 months), whereby three patients relapsed (#2, #3, #8) and two patients (#4, #5) did not respond to therapy. **b** Monitoring of six CML patients with the atypical *BCR-ABL1* transcript e1a2 was performed for a median time period of 8 months (range 5–20 months). All patients showed an unsatisfied molecular response to therapy. **c** Patients (*n* = 6) with the rarely found e14a3 *BCR-ABL1* transcript could be monitored in the median for 24 months (range 11–170 months) and reached deep molecular remission. **d** Four patients harbored the e13a3 *BCR-ABL1* transcript and could be monitored in the median for 19 months (range 17–106 months). After 17 months patient #28 relapsed, but all other remained in deep molecular remission. **e** The double transcript e13a3/e14a3 could be found in two CML patients with a very good response to therapy. The monitoring could be performed over a median time period of 21 months (range 46–61 months). **f** Patients with e8a2 *BCR-ABL1* transcript (*n* = 2) were monitored in the median for 11 months (range 18–38 months) and reached a deep molecular remission

Molecular monitoring of 33 CML patients with atypical *BCR-ABL1* transcripts was performed over time periods ranging from 3 months to a maximum of 14 years (median follow-up 16 months) by qPCR (Fig. 2). A total of 330 samples (2–34 per patient, median 8) were analyzed. Most of the patients carried the e19a2 *BCR-ABL1* transcript (*n* = 12) followed by the fusion gene e1a2 found in six patients. Fusion of another *ABL1* exon could be found in six patients harboring the e14a3 *BCR-ABL1* transcript and four patients with the e13a3 fusion gene. Two patients expressed both e13a3 and e14a3. The rare atypical *BCR-ABL1* transcript e8a2 was detected in two patients and monitored in median for 2 years (range 18–38 months). We also analyzed two follow-up samples of one patient with the e6a2 fusion gene (data not shown).

### The individual molecular response (IMR) level

For patients with atypical *BCR-ABL1* transcripts the application of the international scale is not applicable for the assessment of treatment response due to the use of different PCR primers. Therefore, individual molecular response (IMR) level for CML patients with atypical *BCR-ABL1* transcript were applied. With the IMR, the molecular response to therapy is assessed based on the individual baseline of every patient at diagnosis, i.e. IMR1 means a 1 log reduction from the baseline diagnostic sample for that individual, IMR2 a 2 log reduction, etc.

Eight patients (67%) with the atypical *BCR-ABL1* transcript e19a2 reached an IMR1 3 months after diagnosis (Fig. 2a). During further monitoring six patients achieved a deep molecular remission, which we defined as *a* ≥ 4 log reduction from baseline, with undetectable *BCR-ABL1.* Only two CML patients (17%) with the e19a2 transcript had high *BCR-ABL1* levels and did not reach any IMR level.

Patients with the e1a2 *BCR-ABL1* transcript (*n* = 6) reached no better than IMR1 (50%) or failed to reach IMR1 (50%; Fig. 2b).

Patients with the atypical transcript e14a3 showed a good response and four of six patients (67%) already reached IMR2 or better 3 months after diagnosis (Fig. 2c). After 12 months *BCR-ABL1* was not measurable in all patients.

All four CML patients with the e13a3 transcript showed a rapid decrease of their *BCR-ABL1* levels and two of them (50%) had undetectable *BCR-ABL1* 2 years after diagnosis (Fig. 2d).

The double *BCR-ABL1* transcript e13a3/e14a3 was found in two patients which showed rapidly declining *BCR-ABL1* levels with an IMR4 (patient #29) and IMR2 (patient #30) level after 3 months, respectively (Fig. 2e).

The analyzed patients with the e8a2 *BCR-ABL1* transcript showed a good response and achieved fast IMR1 or better (Fig. 2f).

## Discussion

The hallmark of CML is the presence of the Philadelphia chromosome (Ph) with the associated *BCR-ABL1* fusion gene. Depending on the breakpoints in the two involved genes, different transcripts are generated. Most CML patients express the typical e13a2 (b2a2) or e14a2 (b3a2) *BCR-ABL1* transcripts corresponding to the major breakpoint cluster region (M-*BCR*). Alternate breakpoints in either of both genes generate other rare transcripts. These atypical fusion transcripts are only seen in 1–2% of CML patients (Baccarani et al. 2019) but require particular attention regarding molecular monitoring since they are not covered by routine RT-qPCR methods and might generate false-negative results.

The present study established oligonucleotides and plasmid standards for monitoring atypical *BCR-ABL1* transcripts by RT-qPCR to assess treatment response and residual disease. At first, it is essential to determine the precise rearrangement by multiplex PCR at CML diagnosis prior to the start of TKI treatment (Cross et al. 1994). Otherwise false negative values could be measured and loss of response to therapy could not be detected as recently reported (Sharplin et al. 2019).

Concerning the clinical and hematologic features of e6a2 CML patients it was hypothesized that this CML type represents a different biological entity associated with a worse prognosis (Colla et al. 2004). We measured one patient with the atypical *BCR-ABL1* transcript e6a2 3.7 and 4.2 years after diagnosis and found high *BCR-ABL1/GUSB* ratios of 14.2 and 63.4% respectively, which clearly indicates an inadequate molecular response. Even after allogeneic stem cell transplantation high *BCR-ABL1* levels were detected.

The *BCR-ABL1* transcript e1a2, typically seen in Ph^+^ ALL, is found in approximately 1% of CML patients and has also been associated with poor prognosis. Most of these patients do not achieve a molecular response with tyrosine kinase inhibitor (TKI) therapy and are candidates for stem cell transplantation (Verma et al. 2009). All six patients in our study cohort showed high *BCR-ABL1* levels supporting the evidence of the inferior outcome of e1a2 CML patients described in the literature (Awad et al. 2019).

The fusion of *BCR* with exon a3 of *ABL1* is extremely rare and is found in 0.9% of *BCR-ABL1*-positive patients (Baccarani et al. 2019; Snyder et al. 2004). This fusion transcript lacks part of the SH3 domain of *ABL1* which contributes to leukemogenesis by negatively regulating kinase domain SH1 and activating the STAT5 signaling pathway. Because of an alteration of the tertiary structure of *BCR-ABL1*, the TKI response mechanism is different, but CML patients show a very good response to therapy and have a good prognosis (Duan et al. 2017). In total 36% (12 of 33 patients) of analyzed patients harbored the rearrangement involving a3 either with *BCR* exon 13 (e13a3) or exon 14 (e14a3). Two CML patients showed a double transcript e13a3/e14a3. Almost all patients (83%) achieved deep molecular remission with *BCR-ABL1* values below the detection limit. Only patient #28 with the atypical transcript e13a3 showed increasing *BCR-ABL1* levels corresponding to IMR1 16 month after the diagnosis after the previous achievement of IMR4.

Most patients of the analyzed cohort expressed e19a2 *BCR-ABL1* transcripts (*n* = 12) whereby two patients never achieved IMR1 and three patients lost previously achieved IMR2 or IMR1. Arun et al*.* (2017) observed rapid disease progression, imatinib resistance and blast transformation in CML patients with e19a2 rearrangement.

Whether the fusion gene influences the clinical parameters and outcomes of the CML patients is still a point of discussion (Baccarani et al. 2019; Melo 1996). The study presented here confirms an inadequate molecular response to TKI therapy in patients with e1a2 *BCR-ABL1* transcripts which might therefore be considered as a high-risk group (Awad et al. 2019).

In conclusion, although few patients are diagnosed with atypical *BCR-ABL1* transcripts, their characterization is crucial for proper assessment of treatment response and to avoid false-negative results. We established several RT-qPCR protocols for monitoring all known unusual fusion transcripts, whereby the residual disease of these patients is assessed primarily by considering a decrease of *BCR-ABL1*/*GUSB* ratios over time compared to the initial patient-specific pretreatment sample. qPCR results of patients with atypical *BCR-ABL1* transcripts cannot be reported on the International Scale (IS) and thus the common molecular milestones and guidelines for treatment discontinuation are difficult to apply. We recommend the evaluation of CML patients with atypical *BCR-ABL1* transcripts using the "individual molecular response” (IMR) level presented here.

### EUTOS recommendations

1. At diagnosis, multiplex PCR or equivalent should be performed to detect the underlying *BCR-ABL1* transcript type in all patients with suspected CML.
2. For cases with unusual *BCR-ABL1* transcript sizes direct sequencing should be performed to identify the precise fusion.
3. In the pretherapeutic sample, *BCR-ABL1* should ideally be quantified using *GUSB* as an independent control gene.
4. Molecular monitoring should be performed at the intervals recommended for the specific treatment situation.
5. *BCR-ABL1/GUSB* ratios should be compared with the result from the pretherapeutic sample and expressed as individual molecular response (IMR).
6. In the case of undetectable *BCR-ABL1* IMR response levels are scored based on *GUSB* transcript numbers and similar criteria as for MR scoring on the International Scale (Cross et al. 2015) is applied.
7. Calculation of a result expressed on the International Scale (IS) is not feasible and therefore not recommended.

## Supplementary Information

Below is the link to the electronic supplementary material.

Supplementary file1 (PDF 129 KB)

## Data Availability

All data generated or analyzed during this study are included in this published article and its supplementary information files.
