# Supplementary material for: Assessment of individual molecular response in chronic myeloid leukemia patients with atypical BCR-ABL1 fusion transcripts: recommendations by the EUTOS cooperative network
Source: J Cancer Res Clin Oncol. 2021 Mar 7;147(10):3081–9. doi: 10.1007/s00432-021-03569-8 (PMC8397658; doi:10.1007/s00432-021-03569-8)
Supplement: Supplementary file 1 — Supplementary file1 (PDF 129 KB) [file 432_2021_3569_MOESM1_ESM.pdf]

Assessment of individual molecular response in chronic myeloid leukemia patients with atypical *BCR-ABL1* fusion transcripts. Recommendations by the EUTOS cooperative network.

Journal of Cancer Research and Clinical Oncology

Vivien Schäfer[1], Helen E White[2], Gareth Gerrard[3], Susanne Möbius[1], Susanne Saussele[4], Georg Franke[5], François-X. Mahon[6], Rodica Talmaci[7], Dolors Colomer[8], Simona Soverini[9], Katerina Machova Polakova[10], Nicholas CP Cross[2], Andreas Hochhaus[1], Thomas Ernst[1]

Corresponding Author:
Prof. Dr. Thomas Ernst
Abteilung Hämatologie und Internistische Onkologie
Klinik für Innere Medizin II
Universitätsklinikum Jena
Am Klinikum 1, 07747 Jena, Germany
E-Mail: thomas.ernst@med.uni-jena.de

Figure S1: pUC18_BCR_GUSB

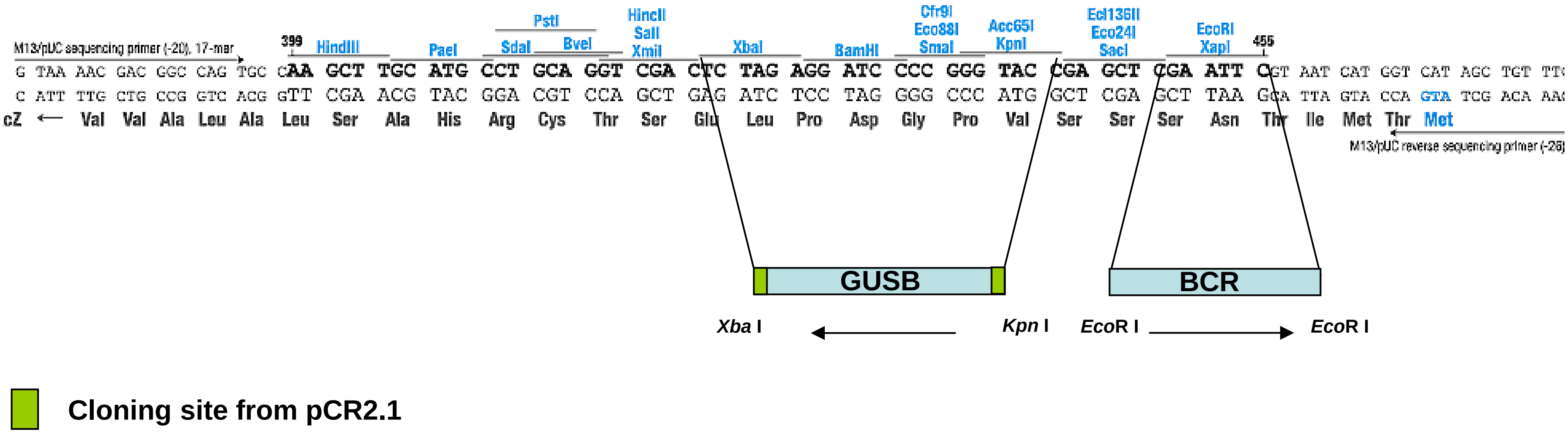

Cloning site from pCR2.1

GUSB: cloned insert (813bp)

TTTCCGTACCAGCCACTACCCCTATGCAGAGGAAGTGATGCAGATGTGTGACCGCTATGGGATTGTGGTCATCGATGAGTGTCCCGGCGTGGGCCTGGCGCTGCCGC
AGTTCTTCAACAACGTTTCTCTGCATCACCACATGCAGGTGATGGAAGAAGTGGTGCGTAGGGACAAGAACCACCCCGCGGTCGTGATGTGGTCTGTGGCCAACGAG
CCTGCGTCCCACCTAGAATCTGCTGGCTACTACTTGAAGATGGTGATCGCTCACACCAAATCCTTGGACCCCTCCCGGCCTGTGACCTTTGTGAGCAACTCTAACTA
TGCAGCAGACAAGGGGGCTCCGTATGTGGATGTGATCTGTTTGAACAGCTACTACTCTTGGTATCACGACTACGGGCACCTGGAGTTGATTCAGCTGCAGCTGGCCA
CCCAGTTTGAGAACTGGTATAAGAAGTATCAGAAGCCCATTATTCAGAGCGAGTATGGAGCAGAAACGATTGCAGGGTTTCACCAGGATCCACCTCTGATGTTCACT
GAAGAGTACCAGAAAAGTCTGCTAGAGCAGTACCATCTGGGTCTGGATCAAAAACGCAGAAAATATGTGGTTGGAGAGCTCATTTGGAATTTTGCCGATTTCATGAC
TGAACAGTCACCGACGAGAGTGCTGGGGAATAAAAAGGGGATCTTCACTCGGCAGAGACAACCAAAAAGTGCAGCGTTCCTTTTGCGAGAGAGATACTGGAAGATTG
CCAATGAAACCAGGTATCCCCACTCAGTAGCCAAGTCACAATGTTTGGAAAACAGCCCGTTTAC

BCR: cloned insert (963bp)

GTCCACTCAGCCACTGGATTTAAGCAGAGTTCAAATCTGTACTGCACCCTGGAGGTGGATTCCTTTGGGTATTTTGTGAATAAAGCAAAGACGCGCGTCTACAGGGA
CACAGCTGAGCCAAACTGGAACGAGGAATTTGAGATAGAGCTGGAGGGCTCCCAGACCCTGAGGATACTGTGCTATGAAAAGTGTTACAACAAGACGAAGATCCCCA
AGGAGGACGGCGAGAGCACGGACAGACTCATGGGGAAGGGCCAGGTCCAGCTGGACCCGCAGGCCCTGCAGGACAGAGACTGGCAGCGCACCGTCATCGCCATGAAT
GGGATCGAAGTAAAGCTCTCGGTCAAGTTCAACAGCAGGGAGTTCAGCTTGAAGAGGATGCCGTCCCGAAAACAGACAGGGGTCTTCGGAGTCAAGATTGCTGTGGT
CACCAAGAGAGAGAGAGGTCCAAGGTGCCCTACATCGTGCGCCAGTGCGTGGAGGAGATCGAGCGCCGAGGCATGGAGGAGGTGGGCATCTACCGCGTGTCCGGTGTGG
CCACGGACATCCAGGCACTGAAGGCAGCCTTCGACGTCAATAACAAGGATGTGTCGGTGATGATGAGCGAGATGGACGTGAACGCCATCGCAGGCACGCTGAAGCTG
TACTTCCGTGAGCTGCCCGAGCCCCTCTTCACTGACGAGTTCTACCCCAACTTCGCAGAGGGCATCGCTCTTTCAGACCCGGTTGCAAAGGAGAGCTGCATGCTCAA
CCTGCTGCTGTCCCTGCCGGAGGCCAACCTGCTCACCTTCCTTTTCCTTCTGGACCACCTGAAAAGGGTGGCAGAGAAGGAGGCAGTCAATAAGATGTCCCTGCACA
ACCTCGCCACGGTCTTTGGCCCCACGCTGCTCCGGCCCTCCGAGAAGGAGAGCAAGCTCCCTGCCAACCCCAGCCAGCCTATCACCATGACTGACAGCTGGTCCTTG

Figure S2: pUC18_BCR_GUSB_ABL1

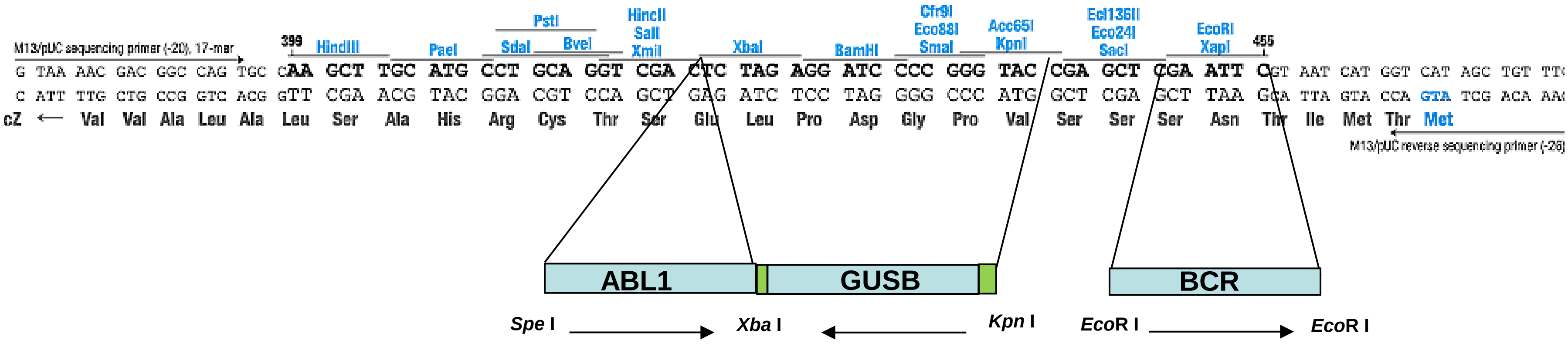

Cloning site from pCR2.1

```
ABL: cloned insert (1803 bp)

CGTTGGAACTCCAAGGAAAACCTTCTCGCTGGACCCAGTGAAAATGACCCCAACCTTTTCGTTGCACTGTATGATTTTGTGGCCAGTGGAGATAACACTCTAAGCAT
AACTAAAGGTGAAAAGCTCCGGGTCTTAGGCTATAATCACAATGGGGAATGGTGTGAAGCCCAAACCAAAAATGGCCAAGGCTGGGTCCCAAGCAACTACATCACGC
CAGTCAACAGTCTGGAGAAACACTCCTGGTACCATGGGCCTGTGTCCCGCAATGCCGCTGAGTATCTGCTGAGCAGCGGGATCAATGGCAGCTTCTTGGTGCGTGAG
AGTGAGAGCAGTCCTGGCCAGAGGTCCATCTCGCTGAGATACGAAGGGAGGGTGTACCATTACAGGATCAACACTGCTTCTGATGGCAAGCTCTACGTCTCCTCCGA
GAGCCGCTTCAACACCCTGGCCGAGTTGGTTCATCATCATTCAACGGTGGCCGACGGGCTCATCACCACGCTCCATTATCCAGCCCCAAAGCGCAACAAGCCCACTG
TCTATGGTGTGTCCCCCAACTACGACAAGTGGGAGATGGAACGCACGGACATCACCATGAAGCACAAGCTGGGCGGGGGCCAGTACGGGGAGGTGTACGAGGGCGTG
TGGAAGAAATACAGCCTGACGGTGGCCGTGAAGACCTTGAAGGAGGACACCATGGAGGTGGAAGAGTTCTTGAAAGAAGCTGCAGTCATGAAAGAGATCAAACACCC
TAACCTGGTGCAGCTCCTTGGGGTCTGCACCCGGGAGCCCCCGTTCTATATCATCACTGAGTTCATGACCTACGGGAACCTCCTGGACTACCTGAGGGAGTGCAACC
GGCAGGAGGTGAACGCCGTGGTGCTGCTGTACATGGCCACTCAGATCTCGTCAGCCATGGAGTACCTGGAGAAGAAAAACTTCATCCACAGAGATCTTGCTGCCCGA
AACTGCCTGGTAGGGGAGAACCACTTGGTGAAGGTAGCTGATTTTGGCCTGAGCAGGTTGATGACAGGGGACACCTACACAGCCCATGCTGGAGCCAAGTTCCCCAT
CAAATGGACTGCACCCGAGAGCCTGGCCTACAACAAGTTCTCCATCAAGTCCGACGTCTGGGCATTTGGAGTATTGCTTTGGGAAATTGCTACCTATGGCATGTCCC
CTTACCCGGGAATTGACCTGTCCCAGGTGTATGAGCTGCTAGAGAAGGACTACCGCATGGAGCGCCCAGAAGGCTGCCCAGAGAAGGTCTATGAACTCATGCGAGCA
TGTTGGCAGTGGAATCCCTCTGACCGGCCCTCCTTTGCTGAAATCCACCAAGCCTTTGAAACAATGTTCCAGGAATCCAGTATCTCAGACGAAGTGGAAAAGGAGCT
GGGGAAACAAGGCGTCCGTGGGGCTGTGAGTACCTTGCTGCAGGCCCCAGAGCTGCCCACCAAGACGAGGACCTCCAGGAGAGCTGCAGAGCACAGAGACACCACTG
ACGTGCCTGAGATGCCTCACTCCAAGGGCCAGGGAGAGAGCGATCCTCTGGACCATGAGCCTGCCGTGTCTCCATTGCTCCCTCGAAAAGAGCGAGGTCCCCCGGAG
GGCGGCCTGAATGAAGATGAGCGCCTTCTCCCCAAAGACAAAAAGACCAACTTGTTCAGCGCCTTGATCAAGAAGAAGAAGAAGACAGCCCCAACCCCTCCCAAACG
CAGCAGCTCCTTCCGGGAGATGGACGGCCAGCCGGAGCGCAGAGGGGCCGGCGAGGAAGAGGGCCGAGACATCAGCAACGGGGCACTGGCT
```

Figure S3: pUC18_BCR_GUSB_e6a2

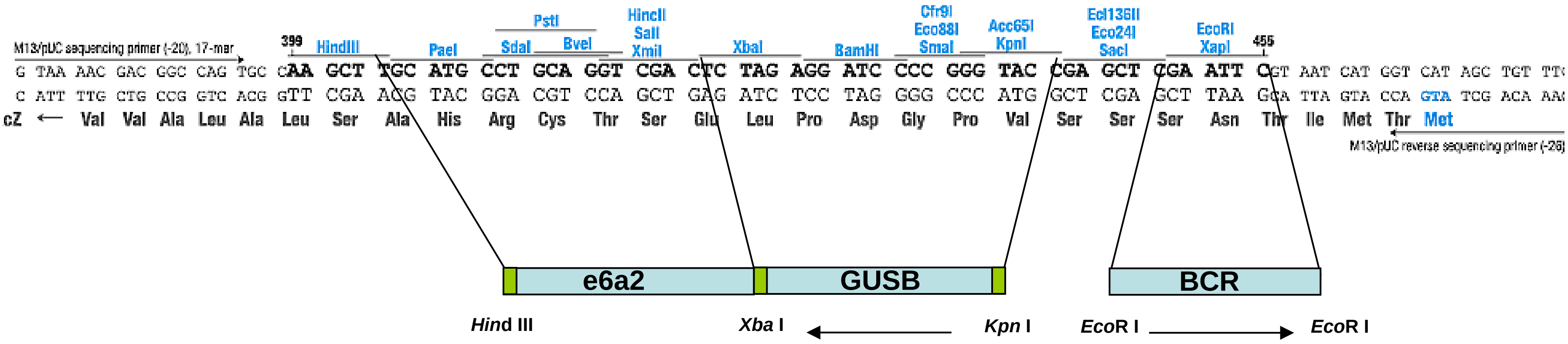

Cloning site from pCR2.1

```
E6a2: cloned insert (1478bp)

CAAAGATGCCAAGGATCCAACGACCAAGAACTCTCTGGAAAAAGCCCTTCAGCGGCCAGTAGCATCTGACTTTGAGCCTCAGGGTCTGAGTGAAGCCGCTCGTTGGA
ACTCCAAGGAAAACCTTCTCGCTGGACCCAGTGAAAATGACCCCAACCTTTTCGTTGCACTGTATGATTTTGTGGCCAGTGGAGATAACACTCTAAGCATAACTAAA
GGTGAAAAGCTCCGGGTCTTAGGCTATAATCACAATGGGGAATGGTGTGAAGCCCAAACCAAAAATGGCCAAGGCTGGGTCCCAAGCAACTACATCACGCCAGTCAA
CAGTCTGGAGAAACACTCCTGGTACCATGGGCCTGTGTCCCGCAATGCCGCTGAGTATCTGCTGAGCAGCGGGATCAATGGCAGCTTCTTGGTGCGTGAGAGTGAGA
GCAGTCCTGGCCAGAGGTCCATCTCGCTGAGATACGAAGGGAGGGTGTACCATTACAGGATCAACACTGCTTCTGATGGCAAGCTCTACGTCTCCTCCGAGAGCCGC
TTCAACACCCTGGCCGAGTTGGTTCATCATCATTCAACGGTGGCCGACGGGCTCATCACCACGCTCCATTATCCAGCCCCAAAGCGCAACAAGCCCACTGTCTATGG
TGTGTCCCCCAACTACGACAAGTGGGAGATGGAACGCACGGACATCACCATGAAGCACAAGCTGGGCGGGGGCCAGTACGGGGAGGTGTACGAGGGCGTGTGGAAGA
AATACAGCCTGACGGTGGCCGTGAAGACCTTGAAGGAGGACACCATGGAGGTGGAAGAGTTCTTGAAAGAAGCTGCAGTCATGAAAGAGATCAAACACCCTAACCTG
GTGCAGCTCCTTGGGGTCTGCACCCGGGAGCCCCCGTTCTATATCATCACTGAGTTCATGACCTACGGGAACCTCCTGGACTACCTGAGGGAGTGCAACCGGCAGGA
GGTGAACGCCGTGGTGCTGCTGTACATGGCCACTCAGATCTCGTCAGCCATGGAGTACCTGGAGAAGAAAAACTTCATCCACAGAGATCTTGCTGCCCGAAACTGCC
TGGTAGGGGAGAACCACTTGGTGAAGGTAGCTGATTTTGGCCTGAGCAGGTTGATGACAGGGGACACCTACACAGCCCATGCTGGAGCCAAGTTCCCCATCAAATGG
ACTGCACCCGAGAGCCTGGCCTACAACAAGTTCTCCATCAAGTCCGACGTCTGGGCATTTGGAGTATTGCTTTGGGAAATTGCTACCTATGGCATGTCCCCTTACCC
GGGAATTGACCTGTCCCAGGTGTATGAGCTGCTAGAGAAGGACTACCGCATGGAGCGCCCAGAAGGCTGCCCAGAGAAGGTCTATGAACTCATGCGAGCATGTTGGC
AGTGGAATCCCTCTGACCGGCCCTCCTTTGCTGAAATCCACCAAGCCTTTGAAACAATGTTCCAGGAATCCAGTATCTCAGACGAAG
```

Figure S4: pUC18_BCR_GUSB_e8a2

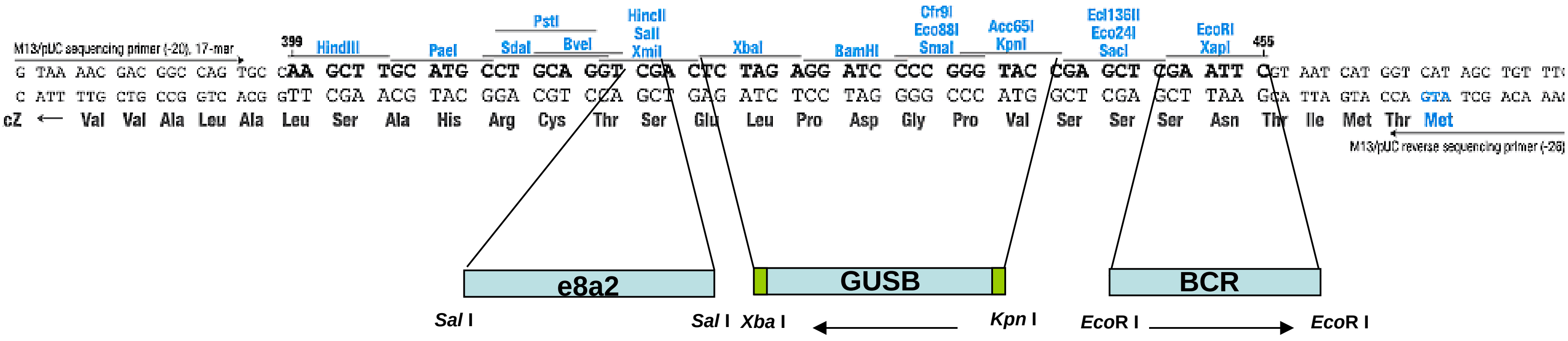

Cloning site from pCR2.1

```
E8a2: cloned insert (973bp)

GTCCTCCATGACTTGCTGAAGCACACTCCTGCCAGCCACCCTGACCACCCCTTGCTGCAGGACGCCCTCCGCATCTCACAGAACTTCCTGTCCAGCATCAATGAGGA
GATCACACCCCGACGGCAGTCCATGACGGTGAAGAAGGGAGAG[GGAGAAGACAGGATGAAAGCTTCATCAACGAGGAAGAGATTACTCCTTATGGAAG]AAGCCCT
TCAGCGGCCAGTAGCATCTGACTTTGAGCCTCAGGGTCTGAGTGAAGCCGCTCGTTGGAACTCCAAGGAAAACCTTCTCGCTGGACCCAGTGAAAATGACCCCAACC
TTTTCGTTGCACTGTATGATTTTGTGGCCAGTGGAGATAACACTCTAAGCATAACTAAAGGTGAAAAGCTCCGGGTCTTAGGCTATAATCACAATGGGGAATGGTGT
GAAGCCCAAACCAAAAATGGCCAAGGCTGGGTCCCAAGCAACTACATCACGCCAGTCAACAGTCTGGAGAAACACTCCTGGTACCATGGGCCTGTGTCCCGCAATGC
CGCTGAGTATCTGCTGAGCAGCGGGATCAATGGCAGCTTCTTGGTGCGTGAGAGTGAGAGCAGTCCTGGCCAGAGGTCCATCTCGCTGAGATACGAAGGGAGGGTGT
ACCATTACAGGATCAACACTGCTTCTGATGGCAAGCTCTACGTCTCCTCCGAGAGCCGCTTCAACACCCTGGCCGAGTTGGTTCATCATCATTCAACGGTGGCCGAC
GGGCTCATCACCACGCTCCATTATCCAGCCCCAAAGCGCAACAAGCCCACTGTCTATGGTGTGTCCCCCAACTACGACAAGTGGGAGATGGAACGCACGGACATCAC
CATGAAGCACAAGCTGGGCGGGGGCCAGTACGGGGAGGTGTACGAGGGCGTGTGGAAGAAATACAGCCTGACGGTGGCCGTGAAGACCTTGAAGGAGGACACCATGG
AGGTGGAAGAG
```

Figure S5: pUC18_BCR_GUSB_e19a2

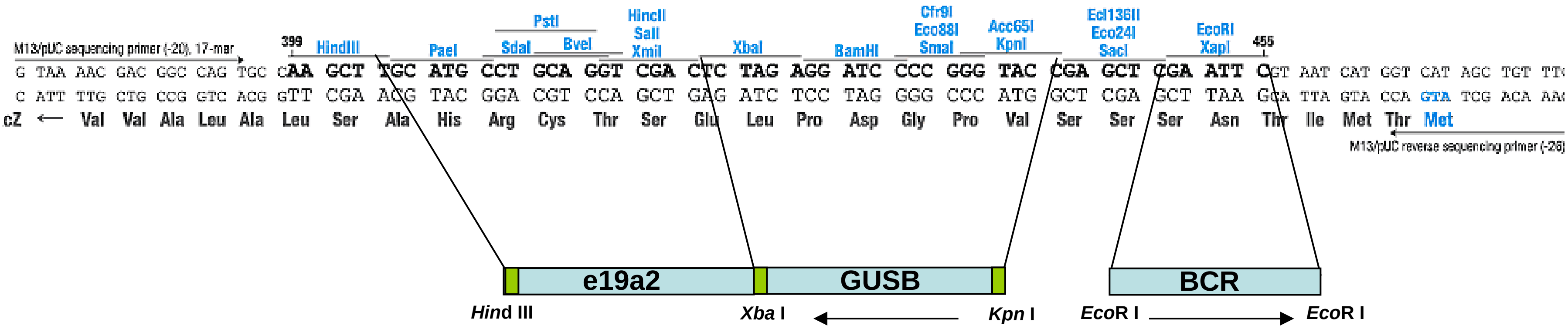

Cloning site from pCR2.1

```
E19a2: cloned insert (1646bp)

GGAGGAGGTGGGCATCTACCGCGTGTCCGGTGTGGCCACGGACATCCAGGCACTGAAGGCAGCCTTCGACGTCAATAACAAGGACGTGTCGGTGATGATGAGCGAGA
TGGACGTGAACGCCATCGCAGGCACGCTGAAGCTGTACTTCCGTGAGCTGCCCGAGCCCCTCTTCACTGACGAGTTCTACCCCAACTTCGCAGAGGGCATCGAAGCC
CTTCAGCGGCCAGTAGCATCTGACTTTGAGCCTCAGGGTCTGAGTGAAGCCGCTCGTTGGAACTCCAAGGAAAACCTTCTCGCTGGACCCAGTGAAAATGACCCCAA
CCTTTTCGTTGCACTGTATGATTTTGTGGCCAGTGGAGATAACACTCTAAGCATAACTAAAGGTGAAAAGCTCCGGGTCTTAGGCTATAATCACAATGGGGAATGGT
GTGAAGCCCAAACCAAAAATGGCCAAGGCTGGGTCCCAAGCAACTACATCACGCCAGTCAACAGTCTGGAGAAACACTCCTGGTACCATGGGCCTGTGTCCCGCAAT
GCCGCTGAGTATCTGCTGAGCAGCGGGATCAATGGCAGCTTCTTGGTGCGTGAGAGTGAGAGCAGTCCTGGCCAGAGGTCCATCTCGCTGAGATACGAAGGGAGGGT
GTACCATTACAGGATCAACACTGCTTCTGATGGCAAGCTCTACGTCTCCTCCGAGAGCCGCTTCAACACCCTGGCCGAGTTGGTTCATCATCATTCAACGGTGGCCG
ACGGGCTCATCACCACGCTCCATTATCCAGCCCCAAAGCGCAACAAGCCCACTGTCTATGGTGTGTCCCCCAACTACGACAAGTGGGAGATGGAACGCACGGACATC
ACCATGAAGCACAAGCTGGGCGGGGGCCAGTACGGGGAGGTGTACGAGGGCGTGTGGAAGAAATACAGCCTGACGGTGGCCGTGAAGACCTTGAAGGAGGACACCAT
GGAGGTGGAAGAGTTCTTGAAAGAAGCTGCAGTCATGAAAGAGATCAAACACCCTAACCTGGTGCAGCTCCTTGGGGTCTGCACCCGGGAGCCCCCGTTCTATATCA
TCACTGAGTTCATGACCTACGGGAACCTCCTGGACTACCTGAGGGAGTGCAACCGGCAGGAGGTGAACGCCGTGGTGCTGCTGTACATGGCCACTCAGATCTCGTCA
GCCATGGAGTACCTGGAGAAGAAAAACTTCATCCACAGAGATCTTGCTGCCCGAAACTGCCTGGTAGGGGAGAACCACTTGGTGAAGGTAGCTGATTTTGGCCTGAG
CAGGTTGATGACAGGGGACACCTACACAGCCCATGCTGGAGCCAAGTTCCCCATCAAATGGACTGCACCCGAGAGCCTGGCCTACAACAAGTTCTCCATCAAGTCCG
ACGTCTGGGCATTTGGAGTATTGCTTTGGGAAATTGCTACCTATGGCATGTCCCCTTACCCGGGAATTGACCTGTCCCAGGTGTATGAGCTGCTAGAGAAGGACTAC
CGCATGGAGCGCCCAGAAGGCTGCCCAGAGAAGGTCTATGAACTCATGCGAGCATGTTGGCAGTGGAATCCCTCTGACCGGCCCTCCTTTGCTGAAATCCACCAAGC
CTTTGAAACAATGTTCCAGGAATCCAGTATCTCAGACGAAG
```

Figure S6: pUC18_BCR_GUSB_ABL1_e14a3

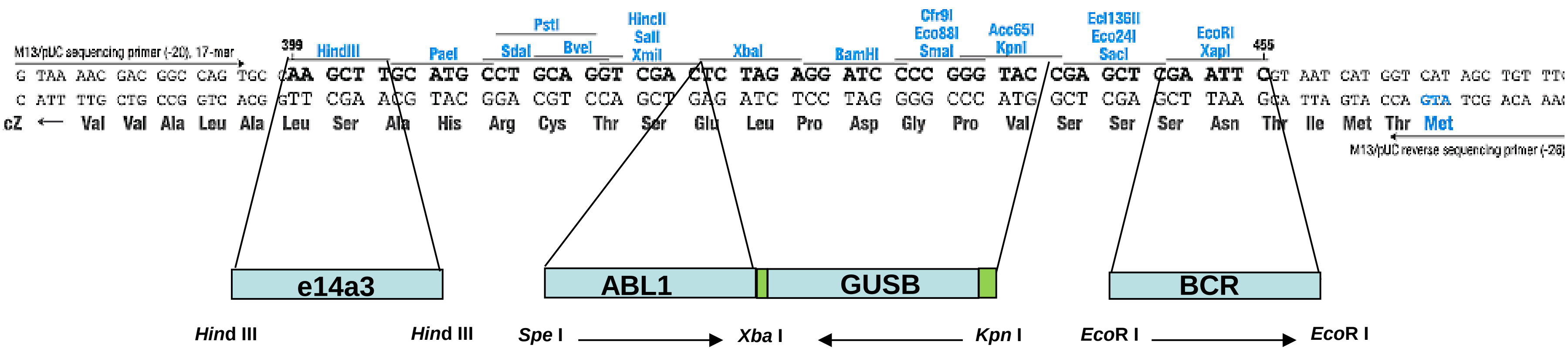

Cloning site from pCR2.1

E14a3: cloned insert (1433bp)

CTTCTCCCTGACATCCGTGGAGCTGCAGATGCTGACCAACTCGTGTGTGAAACTCCAGACTGTCCACAGCATTCCGCTGACCATCAATAAGGAAGATGATGAGTCTC
CGGGGCTCTATGGGTTTCTGAATGTCATCGTCCACTCAGCCACTGGATTTAAGCAGAGTTCAAGTGAAAAGCTCCGGGTCTTAGGCTATAATCACAATGGGGAATGG
TGTGAAGCCCAAACCAAAAATGGCCAAGGCTGGGTCCCAAGCAACTACATCACGCCAGTCAACAGTCTGGAGAAACACTCCTGGTACCATGGGCCTGTGTCCCGCAA
TGCCGCTGAGTATCTGCTGAGCAGCGGGATCAATGGCAGCTTCTTGGTGCGTGAGAGTGAGAGCAGTCCTGGCCAGAGGTCCATCTCGCTGAGATACGAAGGGAGGG
TGTACCATTACAGGATCAACACTGCTTCTGATGGCAAGCTCTACGTCTCCTCCGAGAGCCGCTTCAACACCCTGGCCGAGTTGGTTCATCATCATTCAACGGTGGCC
GACGGGCTCATCACCACGCTCCATTATCCAGCCCCAAAGCGCAACAAGCCCACTGTCTATGGTGTGTCCCCCAACTACGACAAGTGGGAGATGGAACGCACGGACAT
CACCATGAAGCACAAGCTGGGCGGGGGCCAGTACGGGGAGGTGTACGAGGGCGTGTGGAAGAAATACAGCCTGACGGTGGCCGTGAAGACCTTGAAGGAGGACACCA
TGGAGGTGGAAGAGTTCTTGAAAGAAGCTGCAGTCATGAAAGAGATCAAACACCCTAACCTGGTGCAGCTCCTTGGGGTCTGCACCCGGGAGCCCCCGTTCTATATC
ATCACTGAGTTCATGACCTACGGGAACCTCCTGGACTACCTGAGGGAGTGCAACCGGCAGGAGGTGAACGCCGTGGTGCTGCTGTACATGGCCACTCAGATCTCGTC
AGCCATGGAGTACCTGGAGAAGAAAAACTTCATCCACAGAGATCTTGCTGCCCGAAACTGCCTGGTAGGGGAGAACCACTTGGTGAAGGTAGCTGATTTTGGCCTGA
GCAGGTTGATGACAGGGGACACCTACACAGCCCATGCTGGAGCCAAGTTCCCCATCAAATGGACTGCACCCGAGAGCCTGGCCTACAACAAGTTCTCCATCAAGTCC
GACGTCTGGGCATTTGGAGTATTGCTTTGGGAAATTGCTACCTATGGCATGTCCCCTTACCCGGGAATTGACCTGTCCCAGGTGTATGAGCTGCTAGAGAAGGACTA
CCGCATGGAGCGCCCAGAAGGCTGCCCAGAGAAGGTCTATGAACTCATGCGAGCATGTTGGCAGTGGAATCCCTCTGACCGGCCCTCCTTTGCTGAAATCCACCAAG
CCTTTGAAACAATGTTCCAGGAATCCAGTATCTCAGACGAAG

Figure S7: pME3

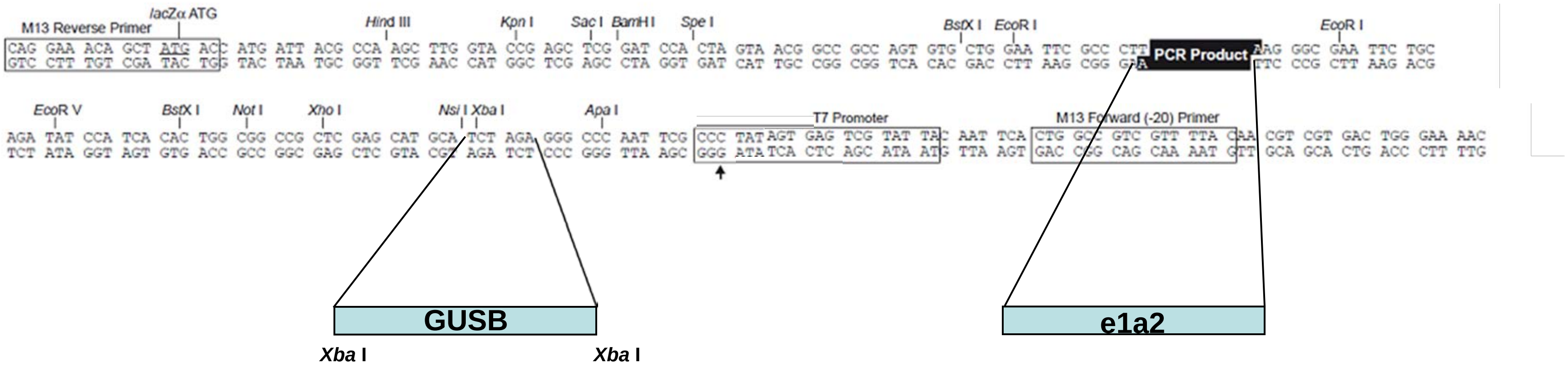

E1a2: cloned insert (870 bp)

AGAACCTCACCTCCAGCGAGGAGGACTTCTCCTCTGGCCAGTCCAGCCGCGTGTCCCCAAGCCCCACCACCTACCGCATGTTCCGGGACAAAAGCCGCTCTCCCTCG
CAGAACTCGCAACAGTCCTTCGACAGCAGCAGTCCCCCCACGCCGCAGTGCCATAAGCGGCACCGGCACTGCCCGGTTGTCGTGTCCGAGGCCACCATCGTGGGCGT
CCGCAAGACCGGGCAGATCTGGCCCAACGATGGCGAGGGCGCCTTCCATGGAGACGCAGAAGCCCTTCAGCGGCCAGTAGCATCTGACTTTGAGCCTCAGGGTCTGA
GTGAAGCCGCTCGTTGGAACTCCAAGGAAAACCTTCTCGCTGGACCCAGTGAAAATGACCCCAACCTTTTCGTTGCACTGTATGATTTTGTGGCCAGTGGAGATAAC
ACTCTAAGCATAACTAAAGGTGAAAAGCTCCGGGTCTTAGGCTATAATCACAATGGGGAATGGTGTGAAGCCCAAACCAAAAATGGCCAAGGCTGGGTCCCAAGCAA
CTACATCACGCCAGTCAACAGTCTGGAGAAACACTCCTGGTACCATGGGCCTGTGTCCCGCAATGCCGCTGAGTATCTGCTGAGCAGCGGGATCAATGGCAGCTTCT
TGGTGCGTGAGAGTGAGAGCAGTCCTGGCCAGAGGTCCATCTCGCTGAGATACGAAGGGAGGGTGTACCATTACAGGATCAACACTGCTTCTGATGGCAAGCTCTAC
GTCTCCTCCGAGAGCCGCTTCAACACCCTGGCCGAGTTGGTTCATCATCATTCAACGGTGGCCGACGGGCTCATCACCACGCTCCATTATCCAGCCCCAAAGCGCAA
CAAGCCCACTGTCT

GUSB: cloned insert (501 bp)

CAACTCTAGAAACGTTCTGGTCTGCCGTGAACAGTCCAGGAGGCACTTGTTCTGCTGCTGTGGAAGTCGCCCTGACTCGGGGAGGAAGGGACACGCAGGTGGTATCA
GTCTTGCTCAAGTAAACAGGCTGTTTTCCAAACATTGTGACTTGGCTACTGAGTGGGGATACCTGGTTTCATTGGCAATCTTCCAGTATCTCTCTCGCAAAAGGAAC
GCTGCACTTTTTGGTTGTCTCTGCCGAGTGAAGATCCCCTTTTTATTCCCCAGCACTCTCGTCGGTGACTGTTCAGTCATGAAATCGGCAAAATTCCAAATGAGCTC
TCCAACCACGTATTTTCTGCGTTTTTGATCCAGACCCAGATGGTACTGCTCTAGCAGACTTTTCTGGTACTCTTCAGTGAACATCAGAGGTGGATCCTGGTGAAACC
CTGCAATCGTTTCTGCTCCATACTCGCTCTGAATAATGGGCTTCTGATACTTCTTATACCAGTTCTAGAACCA

Table S1: Patient characteristic with precise BCR-ABL1 transcript, gender, age at diagnosis and initial tyrosine kinase (TKI) therapy for every patient.

#	***BCR-ABL1* transcript**	**Study**	**Gender**	**Age at diagnosis (years)**	**Initial TKI therapy**
#1	e19a2	CML-IV	m	53	Imatinib
#2	e19a2	CML-IV	m	68	Imatinib
#3	e19a2	CML-IV	m	73	Imatinib
#4	e19a2	CML-IV	m	62	Imatinib
#5	e19a2	CML-IV	m	50	Imatinib
#6	e19a2	CML-IV	m	44	Imatinib
#7	e19a2	CML-V	m	71	Nilotinib
#8	e19a2	ENEST 1st	unknown	33	Nilotinib
#9	e19a2	none	f	52	Dasatinib
#10	e19a2	CML-V	m	66	Nilotinib
#11	e19a2	CML-V	m	78	Nilotinib
#12	e1a2	CML-V	m	75	Nilotinib
#13	e1a2	CML-V	m	56	Nilotinib
#14	e1a2	CORNEA	f	71	Imatinib
#15	e1a2	BYOND	f	67	Bosutinib
#16	e1a2	CML-V	f	75	Nilotinib
#17	e1a2	DasaHit	f	64	Dasatinib
#18	b3a3	ENEST 1st	unknown	unknown	Nilotinib
#19	b3a3	ENEST 1st	unknown	unknown	Nilotinib
#20	b3a3	ENEST 1st	unknown	unknown	Nilotinib
#21	b3a3	IRIS	m	48	Imatinib
#22	b3a3	CML-V	m	54	Nilotinib
#23	b3a3	CML-V	m	28	Nilotinib
#24	b2a3	CML-IV	m	62	Imatinib
#25	b2a3	CML-IV	m	65	Imatinib
#26	b2a3	CML-V	f	51	Nilotinib
#27	b2a3,b3a3	CML-IV	f	50	Imatinib
#28	b2a3,b3a3	CML-IV	m	30	Imatinib
#29	e8a2	CML-V	f	60	Nilotinib
#30	e8a2	none	f	72	Nilotinib
#31	e6a2	EUREKA	m	35	Nilotinib
#32	e8a2	none	f	72	Nilotinib
#33	e6a2	EUREKA	m	35	Nilotinib

TKI tyrosine kinase
